# Access to and experience of education for children and adolescents with cancer: a scoping review protocol

**DOI:** 10.1186/s13643-021-01723-4

**Published:** 2021-06-07

**Authors:** Gemma Bryan, Paula Kelly, Heather Chesters, Jayne Franklin, Helen Griffiths, Loveday Langton, Luke Langton, Claire E. Wakefield, Faith Gibson

**Affiliations:** 1 School of Health Sciences, University of Surrey, Stag Hill, Guildford, GU2 7XH Surrey, UK; 2 Louis Dundas Centre for Children’s Palliative Care, UCL Great Ormond Street Institute of Child Health, 30 Guilford Street, London, UK; 3 Centre for Outcomes and Experience Research in Children’s Health, Illness and Disability (ORCHID), Great Ormond Street Hospital for Children NHS Foundation Trust, 37 Queen Square, London, UK; 4 Great Ormond Street Institute of Child Health Library, UCL Great Ormond Street Institute of Child Health, 30 Guilford Street, London, UK; 5 The Children’s Hospital School at Great Ormond Street Hospital & UCH, Great Ormond Street Hospital for Children NHS Foundation Trust, Great Ormond Street, London, UK; 6 Clinical Health Psychology Psychological Medicine, Oxford University Hospitals NHS Foundation Trust, Children’s Psychological Medicine, John Radcliffe Hospital, Oxford, UK; 7 London, UK; 8 School of Women’s and Children’s Health, UNSW MEDICINE, UNSW, Sydney, Australia; 9 Behavioural Sciences Unit, Kids Cancer Centre, Sydney Children’s Hospital, High Street, Randwick, New South Wales Australia

**Keywords:** Childhood cancer, Education, Schooling, Adolescent cancer

## Abstract

**Background:**

Cancer diagnosis in childhood or adolescence impacts significantly on school attendance, experience and educational outcomes. While there is longstanding recognition in clinical practice that these effects span the whole illness trajectory and continue beyond treatment completion, further clarity is required on the specific barriers and facilitators to education during cancer treatment and beyond, as well as on the experiences of children and adolescents across the full range of education settings (hospital, home, virtual, original school of enrolment), in order to determine which interventions are successful in improving access and experience from their perspective. The aim of this review is to identify what is known from the existing literature about access to and experience of education for children and adolescents with cancer during and post treatment.

**Methods:**

We have planned a scoping literature review searching the following databases from inception onwards: MEDLINE (Ovid), Embase and Embase Classic, Web of Science Core Collection, Education Resources Index, Sociological Abstracts, APA PsycINFO, SCOPUS, CINAHL Plus, Emcare and The Cochrane Library. In addition, DARE, conference abstracts, key journals, and institutional websites will be searched. Arksey and O’Malley’s six-step process will be followed, including a consultation exercise. Studies, reports and policies from any country providing care and treatment for children and adolescents with cancer published in English will be considered eligible for inclusion. Two reviewers will independently screen all citations, full-text articles and abstract data. A narrative summary of findings will be conducted. Data analysis will involve quantitative (e.g., frequencies) and qualitative (e.g., content and thematic analysis) methods.

**Discussion:**

This is a timely examination given the increased incidence of childhood cancer, more intensive treatment regimens and improved survival rates for childhood cancer. The inclusion of a substantive consultation exercise with families and professionals will provide an important opportunity to examine the scoping review outputs. Findings will assist the childhood cancer community in developing a comprehensive evidence-based understanding of a significant associated bio-psychosocial impact of cancer diagnosis and treatment and will form the first step towards developing effective interventions and policies to mitigate identified detrimental effects.

**Systematic review registration:**

Open Science Framework (osf/io/yc4wt)

**Supplementary Information:**

The online version contains supplementary material available at 10.1186/s13643-021-01723-4.

## Background

A paediatric cancer diagnosis can affect a child’s school experience throughout the entire treatment trajectory [1]. At diagnosis and during treatment, absenteeism due to therapy regimens and their associated side-effects can lead to increased social difficulties and feelings of disconnection, inhibiting the child’s ability to learn and engage in their education [1–3]. Post-treatment, absenteeism may continue, and permanent sensory and neurocognitive deficits may impact a child’s ability to engage in school and consequently affect their academic achievements [1, 3, 4]. As many as 30% of survivors of childhood cancer are forced to repeat a year of schooling due to the interruptions experienced [3, 5, 6]. While social isolation may continue to be an issue despite successful engagement with school [7].

There has been widespread recognition of the impact of cancer diagnosis and treatment on educational attainment, with studies documenting that survivors perform less well overall [3, 8]. A recent systematic review drew attention to the association between educational attainment, emotional well-being and economic security, as an imperative for actioning educational support for survivors [9]. Importantly, their results indicated that a long-standing focus on the under-performance and need for additional educational support for children with central nervous system (CNS) disease and or directed treatment has potentially led to less recognition of the educational challenges for all types of childhood cancer diagnosis and treatment [9]. Of note, the studies identified in the review drew on retrospective data for survivors treated over a significant timespan (1940–2009) which encompass significant changes in treatment protocols, prognosis and associated morbidity for many types of childhood cancer.

Parental concern for their child’s education following diagnosis has also been documented, including noting reductions in academic performance, ability to participate in school and school-related activities and the need for education professionals and school peers to receive information specific to their child’s needs [1]. Education and health care professionals also identify their own needs for training to support the educational needs of children and adolescents with cancer [10]. In England, the Department for Education provides guidance on the statutory obligations that schools have to meet the educational support needs for children with medical conditions, including cancer [11, 12]. Although the need for planning and resources is widely recognised, families and advocacy groups continue to report under use of the guidance [13].

There is a wide range of materials, print and online, providing information on the impacts of cancer treatments on educational experience. However, online resources have been found to be highly variable in quality of content and to have limited accessibility for the majority of parents [14]. Securing appropriate educational support for their child is an additional advocacy role for parents of children with cancer, requiring understanding of the educational systems and relying on parents to act as brokers of information between health care and educational services [15, 16]. This may be required throughout their child’s educational career—often extending many years beyond treatment completion.

A range of interventions have been developed to support children with cancer in accessing education during and beyond treatment [2]. These include access to education through hospital-based school services and initiatives aimed at maintaining contact with their own school peers and teachers during hospitalisation; home schooling programmes and web-based technology links to the child’s original school of enrolment [17]; and school re-entry programmes [18] which provide information and support to education professionals, parents, school peer groups and patients about the impact of cancer on learning and the social, emotional and practical factors of returning to school. These interventions may include how to access statutory and specialist educational services to ensure long-term assessment and individual input for survivors throughout their educational career. Studies reporting these approaches are often small scale, linked to a single children’s oncology centre, with no planned strategy for mandating long-term provision [19].

There is limited recent and robust evidence regarding which interventions have a positive effect on children and adolescents access to and experience of education during care and treatment for cancer. Thompson et al. [19] identified 17 peer-reviewed papers focused on school re-entry support to maintain academic continuity and highlighted the lack of data related to children’s own perspectives and those of their parents of interventions. A later review also concluded that the typically low quality of studies precluded their ability to recommend a specific intervention approach related to school re-entry [4].

Children’s own experiences appear to have received insufficient investigation given the extensive concerns related to educational attainment. Retrospective reporting [20] and small sample sizes [21] are further issues that challenge the drawing together of robust recommendations from individual studies. Children’s own perceptions seem to have a strong focus on the social aspects of school life [22]; these can form both the positive aspects of school and some of the more challenging aspects as reported by children and adolescents themselves [23]. Children’s concerns regarding their academic performance often appear secondary; however, the limited data reviewed to date may have failed to reveal variations regarding for example specific cancer diagnosis, stage of schooling, school orientation to academic performance and previous school experience.

There is therefore a need to better understand the associated bio-psychosocial impact of diagnosis and treatment for children and adolescents, in the context of the increasing incidence of childhood cancer and improvements in survival (particularly in high-income countries). Only through improved understanding and effective interventions to mitigate identified detrimental effects can the full benefits of survival be realised by children, adolescents and their families.

This scoping review will examine the published evidence on access to education from diagnosis to beyond treatment completion in all settings and seeks to identify the experiences of children and adolescents, their parents, education and health care professionals to determine how further research can optimise educational opportunities and facilitate effective participation for children and adolescents with cancer. Ethnographic theory will inform our examination of the literature in recognition that school plays a fundamental role in children’s cultural and social lives [24, 25]. Moreover, children will continue to be part of the structures and cultures of health care services throughout and post treatment. Ethnography can provide a framework to analyse these contexts for children, families and the services that support them. At diagnosis and during treatment, children and their families are rapidly socialised into the rules of cancer care and treatment rules-that they are required to follow within the hospital and beyond [26]. In considering a resumption of schooling, they need to integrate the culture of cancer care into education-focused systems. In a similar way, health care professionals, parents and children are required to translate health implications to educate their education professionals, who will in turn interpret these for operating within their own structures.

In mapping the available research knowledge on access and experience of education for children and adolescents with cancer, we will be explicit in recognising the cultural worlds of illness and schooling. In this way, our understanding of current evidence and gaps in knowledge will pave the way for further collaborative and translational research in health care and education to improve educational access and experience for children and adolescents with cancer. This approach will enable their full social and learning potential to be realised.

The objective of this scoping review is to determine the current evidence and gaps in the knowledge base on access to and experience of education for children and adolescents with cancer. In consultation with children, adolescents, parents, health care and education professionals we will use these findings to develop an initiative for further research and interventions to inform and improve practice.

## Methods/design

The review protocol has been registered within the Open Science framework database (osf/io/yc4wt) and is being reported in accordance with the reporting guidance provided in the Preferred Reporting Items for Systematic Reviews and Meta-Analyses Protocols (PRISMA-P) statement [27, 28] (see checklist in Additional file 1). The proposed scoping review will be reported in accordance with the reporting guidance provided in the Preferred Reporting Items for Systematic Reviews and Meta-analyses (PRISMA) extension for Scoping Reviews (PRISMA-ScR) [29]. The scoping review methodology will be conducted in accordance with the framework proposed by Arksey and O'Malley [30] and includes a consultation phase.

This scoping review aims to identify all of the relevant literature—regardless of study design with the aim to identify key concepts and gaps in the literature.

This examination will take an iterative approach, enabling a reflexive review of the search strategy and further refining of the research question as part of the process [31]. The flexibility of the scoping approach will enhance our ability to explore this complex topic. The six steps to be employed in this scoping review include (1) defining the research question; (2) identifying relevant articles; (3) study selection; (4) charting the data; and (5) collating, summarising and reporting the results; and (6) a consultation exercise [30].

### Step 1: defining the research question

The aim of this scoping review is to identify what is known from the existing literature about the access to and experience of education for children and adolescents with cancer during and post treatment.

This will be achieved by answering the following questions:

1. What are the experiences of children and adolescents with cancer regarding their education during and post treatment?
2. What are the experiences of parents, education and health care professionals of the provision of education during and post treatment to children and adolescents with cancer?
3. What are the factors that impact access to education (school/preschool/nursery/college, home schooling, hospital schooling) for children and adolescents with cancer during and post treatment?
4. What are the types, effects and outcomes of interventions to facilitate access to education, including school re-entry and return-to-school programs for children and adolescents with cancer during and post treatment?

### Step 2: identify relevant literature

Literature will be identified through searching the following databases from inception onwards: MEDLINE (Ovid), Embase and Embase Classic, Web of Science Core Collection, Education Resources Index, Sociological Abstracts, APA PsycINFO, SCOPUS, CINAHL Plus, Emcare, The Cochrane Library, and the Database of Abstracts of Reviews of Effects (Table 1: electronic databases to be searched). These databases have been selected as a source of evidence on the research topic involving the academic, policy and practice fields of child health, cancer care and treatment, education and psychosocial well-being for children and families. In addition, we will search the proceedings of the following national and international conferences in these fields from the last 10 years: American Society of Pediatric Haematology and Oncology (ASPHO), Association of Pediatric Haematology and Oncology Nurses (APHON), International Society of Paediatric Oncology (SIOP), Nordic Society for Pediatric Oncology Nurses (NOBOS), Nordic Society of Paediatric Haematology and Oncology (NOPHO) and the Conference Proceedings Citation Index (Table 2: conference abstracts to be searched). Outputs from the last 10 years from the following key journals will also be searched: Cancer Education, Cancer Nursing, Clinical Journal of Oncology Nursing, The Educational and Developmental Psychologist, European Journal of Cancer Care, European Journal of Cancer, European Journal of Oncology Nursing, Journal of Adolescent and Young Adult Oncology, Journal of Cancer Education, Journal of Cancer Survivorship, Journal of Pediatric Oncology Nursing, Pediatric Haematology and Oncology, Pediatric Blood and Cancer, Psycho-oncology, Seminars in Oncology Nursing, Seminars in Oncology and Supportive Care in Cancer (Table 3: key journals to be searched) and the reference lists of all potential inclusions will be hand searched.

**Table 1:** Electronic databases to be searched

Electronic databases (Inception to 2020)	
MEDLINE(R) and Epub Ahead of Print, In-Process, In-Data-Review & Other Non-Indexed Citations and Daily 1946—(Ovid)	
Embase and Embase Classic 1947—(Ovid)	
Web of Science Core Collection	
ERIC—Education Resources Index 1966—(EBSCOhost)	
Sociological Abstracts 1952—(ProQuest)	
APA PsycINFO 1806—(Ovid)	
SCOPUS	
CINAHL Plus—Cumulative Index to Nursing & Allied Health 1937—(EBSCOhost)	
Emcare 1995—(Ovid)	
Cochrane Library	
DARE—The Database of Abstracts of Reviews of Effects 1994-2015 https://www.crd.york.ac.uk/CRDWeb/

**Table 2:** Conference abstracts to be searched

Conference abstracts	
American Society of Pediatric Haematology and Oncology (ASPHO) http://aspho.org/	
Association of pediatric haematology and oncology nurses (APHON) http://conference.aphon.org/	
International Society of Paediatric Oncology (SIOP) https://siop-online.org/	
Nordic Society for Pediatric Oncology Nurses (NOBOS) (Denmark, Iceland, Finland, Norway and Sweden) https://nobos.org/cms	
Nordic Society of Paediatric Haematology and Oncology (NOPHO) http://www.nopho.org/

**Table 3:** Key journals to be searched

Key journals	
Cancer Education	
Cancer Nursing	
Clinical Journal of Oncology Nursing	
The Educational and Developmental Psychologist	
European Journal of Cancer Care	
European Journal of Cancer	
European Journal of Oncology Nursing	
Journal of Adolescent and Young Adult Oncology	
Journal of Cancer Education	
Journal of Cancer Survivorship	
Journal of Pediatric Oncology Nursing	
Pediatric Hematology and Oncology	
Pediatric Blood and Cancer	
Psycho-Oncology	
Seminars in Oncology Nursing	
Seminars in Oncology	
Supportive Care in Cancer	

Cancer as a disease occupies a particular cultural standing which is reflected in the wide range of professional and charitable organisations conducting research, providing information and support to people with cancer and their families. We will explore the website of these key organisations and charities in our search strategy: American Childhood Cancer Organisation, Australian Government Cancer Australia, Australian Research Alliance for Children and Youth, Canadian Cancer Society, Cancer Research UK, Child Cancer Foundation New Zealand, Children’s Cancer and Leukaemia Group (CCLG), Childhood Cancer International (CCI), CLIC Sargent, Macmillan Cancer Support and Teenage Cancer Trust. Our multidisciplinary team has identified three organisations focused on supporting children with medical needs in the education setting and we plan to explore these websites in addition (Medical Needs in Schools, Missing School, National Association for Hospital Schools) (Table 4: organisational websites to be searched).

**Table 4:** **Organisation websites to be searched**

Organisations	
American Childhood Cancer Organisation	
Australian Government Cancer Australia	
Australian Research Alliance for Children and Youth	
Canadian Cancer society	
Cancer Research UK	
Child Cancer Foundation New Zealand.	
Children’s Cancer and Leukaemia Group (CCLG)	
Childhood Cancer International (CCI)	
CLIC Sargent	
Macmillan Cancer Support	
Medical Needs in Schools (UK)	
Missing School (Australia)	
National Association for Hospital Schools (UK)	
Teenage Cancer Trust	

The expertise of an Information Specialist (HC) has been included to identify and develop appropriate search terms to achieve a comprehensive search strategy of key words and subject headings to achieve the largest number of relevant papers.

Specific Medical Subject Headings (MeSH) and free text terms for child, adolescent and related terms such as “school child” or “teen” will be combined with terms for cancer including cancers specific to children. These terms will be combined with MeSH terms for education, teaching and school, key words for example “home school”, “school re-entry”, “hospital school” using Boolean logic operators (and, or) (Additional file 2: Draft search strategy for Medline).

A pilot of the search terms has been conducted in OVID Medline by two members of the research team (PK, GB) to check and refine the initial terms and the revised strategy will then be applied to all of the databases and other source materials. Following the iterative approach, the study team will determine and document the processes undertaken to undertake revised searches as changes are made throughout the study.

The scope of the study will include literature that is readily available in the English language. No restrictions on date of publication will be employed. The initial searches will be run by HC and results imported into Endnote reference management system, duplicate copies will be removed. All titles and abstracts will be independently screened by PK and GB to identify eligible studies. Any disagreements on inclusion/exclusion will be taken to a third reviewer (FG). The number of studies included and excluded at each stage, along with reasons will be summarised in a PRISMA flow diagram, with a record of the rationale for decisions recorded on a Microsoft Office Excel spreadsheet. PK and GB will independently review the full text of all studies and reports identified for inclusion, 10% of the sample will be checked by FG for agreement, and the team will meet to discuss those selected for inclusion. In keeping with scoping review approaches methodological quality will not be assessed.

### Step 3: study selection

To reflect the objectives of this scoping review, we have followed the recommendations of Peters and colleagues [32], defining participants, concepts and context in an explicit way. Participants will include children and adolescents with any cancer diagnosis, acknowledging that the type of cancer and its associated treatment modalities may influence educational experience and access. As children’s and adolescents’ access to educational input is mediated by adults, including their parents, heath care professionals responsible for their care and treatment and education professionals with responsibility for educational provision and or policy, these groups will also be included in the review.

We have identified two key concepts in relation to education for children and adolescents with cancer—access and experience. We have defined access as the opportunities or barriers for children and adolescents with cancer to take part in the anticipated experiences of education across the compulsory education spectrum—which we define as from preschool up until university or other post compulsory education prior to their 19th birthday. Access is taken to include all planned aspects of the curriculum, including participating in academic, social, pastoral, physical learning, and assessments; socialising with peers and extra-curricular activities. We include access to educational opportunities in all settings: hospital school, home-schooling and original school of enrolment reflecting our practice knowledge of the range of settings in which children and adolescents with cancer may receive educational input across their illness trajectory. Since the specific timing for re-engagement with educational services seems to have received relatively little attention in research or practice, we are interested in access during treatment and post treatment. The varied diagnosis and treatment modalities are anticipated to be factors associated with access to education—for example children with acute lymphoblastic leukaemia (the most common type of childhood cancer) have a prolonged period (up to 3 years) of maintenance (lower intensity) therapy. Health care practitioners report return to original school of enrolment is often achieved during this period. For all children and adolescents, the intensity, treatment setting and associated treatment effects on well-being and capacity to access learning may be very variable and require a highly individualised approach. In addition, the bridging from hospital school, home-based schooling programmes to original school of enrolment may be a further factor for access.

The second concept for investigation through this scoping review is experience, which includes the perspectives of children and adolescents of all educational interventions they have received and their experiences of the absence of educational inputs. Recognising the overlap of the concepts of access and experience, we will include the experiences of the participant adults (parents, health care and education professionals) in facilitating and providing education for children and adolescents with cancer.

Finally, the context for this review will include studies, reports and policies from any country providing care and treatment for children and adolescents with cancer, which are published in English, as we are interested to obtain the broadest range of practices reported. In addition, we are aware of the strong international collaboration on clinical treatments for childhood cancer. We reflect the potential for psychosocial care including educational interventions to be shared in the same way.

#### Eligibility criteria

##### Inclusion criteria

Inclusion criteria are studies, reports and policies that

1. Include
  - Children and adolescents under 19 years old with a cancer diagnosis currently receiving treatment or post treatment completion, or
  - Parents of children with cancer who are under 19 years old with a cancer diagnosis currently receiving treatment or post treatment completion, or
  - Health care professionals—any professional providing care and treatment to children and adolescent with cancer in any setting (Specialist children’s cancer centre, local hospital, home, community and primary health, education), or
  - Education professionals—teachers, trainers, teaching assistants, school support workers, and special education staff, or
2. Focus on or include
  - Access to education in any format or setting for children and adolescents with cancer during and post treatment, or
  - The educational experiences of children and adolescents with cancer during and post treatment, or
  - The experiences of parents, and or health care and or education professionals in relation to facilitating access to education for children and adolescents with cancer during and post treatment, or
  - The experiences of parents, and or health care and or education professionals in relation to the ongoing provision of education for children and adolescents with cancer during and post treatment, or
  - Interventions to investigate and support access to education for children and adolescents with cancer during and post treatment, or
  - Interventions to investigate and improve the experiences of education in any setting or format for children and adolescents with cancer during and post treatment.

##### **Exclusion criteria**

Exclusion criteria are studies, reports and policies that

1. Only include
  - Adolescents diagnosed with cancer post compulsory school age (over 19 years) or their parents
  - Children under the age of 3 years diagnosed with cancer and currently receiving treatment or their parents
  - Parents of children with other long-term illness and or life-threatening conditions
  - Heath care professionals providing care or working in settings focused on the care and treatment of children with chronic and complex conditions other than cancer
  - Education professionals and educational provision focused on the care and treatment of children with chronic and complex conditions other than cancer
  - Education professionals addressing the educational needs of young people in post compulsory educational settings, including universities and apprentice courses
2. Are published in languages other than English
3. Focus exclusively on the educational attainment, performance or status of children and adolescents with cancer post treatment
4. Focus on cancer prevention through educational interventions in school or college settings
5. Focus on children and adolescents learning about their cancer diagnosis, care and treatment through educational interventions
6. Studies, reports and policies that focus on parents learning about their child’s or adolescent’s cancer diagnosis, care and treatment through educational interventions

##### Types of studies to be included

The scoping review will include empirical studies with quantitative, qualitative and or mixed methods data, published in English [33]. The review will include book chapters and conference proceedings. Guidelines, reports and policies produced by key childhood cancer societies, charities, Government and health authorities will be included. Information from blogs and social media accounts will be excluded.

### Step 4: charting the data

We will chart the following data (where available) for all included publications into a Microsoft Excel file: the year of publication, country of origin, number, type and age range of participants, type of study, methodology, methods and theoretical orientation. For each paper, the study team will identify further factors to be charted. For example, childhood cancer diagnosis, stage of treatment and duration, number of years on or post treatment, school stage, intervention type and comparator if used, target and setting of intervention, mode of delivery, outcomes and measures used, and key findings as related to the review questions. Data about any cost and sustainability of services and the reach of intervention—national, regional, or single children’s cancer centre—will also be collected. Individual quotations, verbatim text and field notes will be selected with the respondents’ basic demographics, age, gender, diagnosis and treatment stage, where appropriate.

One team member will extract the data (PK), which will be checked by another member of the research team (GB). If there is disagreement between reviewers, a third author (FG) will review and a majority decision will be taken. Missing data will be highlighted. Attempts will be made to contact study authors by email, if necessary, to seek further information or clarification about their population, study design or results.

Data extraction forms will be designed by the research team and piloted on five studies from a varied selection. The format of the forms will be reassessed and revised if required to ensure all relevant data are being captured in a systematic and transparent way.

### Step 5: collating, summarising and reporting the results

We will use a narrative synthesis approach to managing the data—with the aim to summarise and explain the findings of these studies. This will include a numeric overview of all literature findings included in the review, thematic analysis of qualitative data and summary statistics of intervention studies to summarise outcomes.

We will begin by creating a textual summary of data for each study with separate tables for qualitative studies, mixed methods studies, qualitative studies and policy/guidance documents. These summaries and their content will be reviewed by the research team to ensure that the planned approaches to developing a narrative synthesis from the range of literature included is robust and will result in a summary and explanatory narrative for a comprehensive and coherent presentation to the consultation group.

Basic numerical data will be analysed using descriptive statistics in Microsoft Excel software to show relevant frequencies and summarise the literature including trends in years of publication, percentages of studies per country, participant numbers per study (and total numbers), the breakdown of key groups with associated relevant factors, children and adolescents (age, diagnosis, education history), parents, education and health care professionals. Graphical illustrations will show the frequency of methods and methodologies used and the types, settings and outcomes of interventions.

Intervention studies will be reviewed as a group to determine if there are opportunities to assess outcome effects where sufficiently similar outcomes have been measured to warrant analysis of pooled effect. In the same way, types of interventions will be examined to explore the opportunities to interrogate further the impact of particular intervention types if the studies selected report a number of sufficient homogenous interventions. Initial examination of the literature in the field suggests that this may not be possible.

The authors will work together to analyse the narrative texts, quotations and field notes thematically using a constant comparative ethnographic approach as described by Emerson et al. [34] to ensure that contextual issues remain integrated in the analysis, this will be supported by qualitative data analysis software NVivo [35]. There will be an ongoing reflective component to the analysis by continuing to ask ourselves how our preconceptions are influencing this analytical process. The resultant analysis will consist of themes from the data illustrated by data extracts including quotations.

Thematic analysis of individual studies will be synthesised to map the interrelationships between children, parents, education and health care professionals to illustrate how the contexts of home, school and health care institutions shape access and experience of education for children and adolescents with cancer [36]. Visual data will be analysed using content analysis [37]. Data will be presented in tabular, diagrammatic and descriptive summaries. Summarising and identifying patterns in the data from the selected literature, we will create visual mapping documents and textual material including quotations for use in the consultation exercise.

### Step 6: consultation exercise

We regard the inclusion of a consultation exercise as an essential planned component in this review. Recognising the unique and critical perspectives that service users and providers will bring to an examination of the published literature, as Manning et al. [38] have demonstrated in the environment of paediatric intensive care. The aim of the consultation exercise will be to review and discuss the findings of the scoping review in order to make recommendations for further research and dissemination. This consultation exercise will comprise focus groups and individual interviews and will provide an opportunity to check the validity of the findings and further elaborate their relevance for children and adolescents with cancer, their families and key professional groups.

The consultation exercise will be held as a hybrid physical and virtual event. Two face-to-face consultation events will be held in the UK in London and Newcastle, while an unrestricted number of online teleconference calls will take place via Zoom.

The locations selected host two of the UK’s largest paediatric oncology principal treatment centres, treating over 500 patients per year. Each centre provides treatment for all types of childhood malignancies providing a full range of treatment modalities up to the age of 24 years including long-term follow-up post treatment. In addition, the populations that access these centres are diverse in their ethnic, cultural, social and economic background. Access to interpreting and advocacy services to facilitate participation will be sourced [39]. Invited participants will be children at multiple stages of their treatment trajectory and their parents. Children will be included even if they have yet to have the opportunity to return to their original school of enrolment as well as those who have experienced school attendance following treatment.

All participants will be given a choice regarding how they would like to engage in the exercise. Children can elect to participate with their parents or in a separate children’s event at the physical venue or online. Two groups will be invited to take part: children and their families and professional groups delivering care, treatment and education to children with cancer. Children and adolescents previously or currently being treated for cancer together with their siblings and parents will be invited to take part. Families will be asked to identify and invite other members of their extended families and friendship circle who have had a role in the education of their ill child or their siblings. Invites for families will be sent to families via the primary treatment centre, circulated on social media and cascaded via children’s cancer charities.

Paediatric oncology health care professionals (including doctors, nurses, allied health care professionals, psychologists and social workers) and education professionals (including hospital school teachers, home school services, community school teachers, specialist educational support services) will be identified and invited to participate in focus groups. Further professional and stakeholder groups will be invited if the results of the study identify their roles as central factors in access and experience of education for children and adolescents with cancer.

The face-to-face sessions will operate on a ‘drop-in’ session basis with members of the research team available all day to meet with participants. Large gatherings will be avoided and social distancing rules will be followed. As we know that families do not want to make extra trips to the hospital, the face-to-face sessions will be held at accessible neutral venues away from the hospitals (pizza restaurants). Childcare and catering will be provided for attendees.

In both face-to-face and virtual sessions, child and adolescent participants, parents and professionals will be allocated to separate small focus groups for review and discussion of the results of the scoping review. Members of the research team will present a summary of the key findings to each group—this will include quotations from the qualitative literature and facilitate a discussion with participants on how these relate to their own experiences. Child and adolescent participants will be told about the results of the study, and the research team will prepare age-appropriate materials and activities to enable their views to be understood and captured [40, 41]. All participants will be asked to identify challenges and priorities for future research on education for children with cancer during and post therapy.

With the permission of all participants, the focus group discussions will be audio-recorded and transcribed verbatim. In addition, the authors will make notes from the focus groups and write up a reflective log immediately following the group. We will consult with the focus group participants on the dissemination approaches for the study to ensure that these include feedback to relevant stakeholder groups in addition to publishing the findings from the scoping review (incorporating the consultation exercise) in addition to presentation at conferences and publication in peer reviewed journals.

The consultation exercise will follow the principals of research ethics committee and information governance practice. Travel will be paid for all participants who take part in the face-to-face sessions. All participating family members will receive a £30 gift voucher as a thank you for taking part. This consultation exercise with families and professionals will provide an important space to examine the outputs from our scoping review: deemed a valuable approach to enrich the data and to increase the relevance of the results of a scoping review for policy and practice.

## Discussion

A diagnosis of childhood cancer has a wide-ranging and lifelong impact for both the patient and their family. The necessity of receiving treatment leads to breaks from school and even after treatment, absenteeism may continue to be an issue. Treatment-related side effects, such as cognitive deficits and fatigue can make acquisition and retainment of information difficult for some children and adolescents, and leading to lower educational attainment. Despite this, the educational experiences of children and adolescents during and after treatment has received comparatively little attention.

To our knowledge, no previous scoping reviews have been conducted on this topic. This review will bring together the literature about the access to and experience of education for children and adolescents with cancer and their families. As access to educational input is mediated by adults, parents, health care and educational professionals will also be included in this review.

While this scoping review will follow a rigorous method, we anticipate limitations to this review. Firstly, this scoping review is limited to outputs published in English, which may limit the completeness of the findings. However, the broad nature of the research questions, together with extensive searching of key journals, abstracts from major paediatric oncology conferences and cancer organisations, will still allow capture of a significant proportion of the literature available on the experiences of education for children with cancer. Secondly, since this is a scoping review, quality appraisals of included studies will not be conducted. It therefore cannot be determined whether they provide robust and generalisable findings. However, the inclusion of a consultation exercise will enhance the results of the review by incorporating the views of current service users and providers.

Any amendments to this protocol made during the review process will be recorded by the authors, together with the reasons for amendment. This information will be included when reporting the results of this scoping review. The results of this scoping review will be disseminated widely. In addition to traditional approaches to dissemination including published papers in open access health and education journals and presentations at both professional and lay conferences, a report will be distributed to all who have been involved in the consultation exercise, and other relevant stakeholders including established national childhood cancer networks for medicine nursing and allied health staff working in hospital and community settings. Educators who participate in the study will be asked for recommendations of local and national networks to include in distributing findings as widely as possible; recognising that those who attend our planned events, are likely to have been already involved in the care of children with cancer in their current education roles. This report will be produced in a variety of formats, including producing short reports in languages other than English, infographics and short videos. Both paper and online versions of our results will be produced. Social media approaches to dissemination will be maximised where appropriate.

## Supplementary Information

**Additional file 1.** PRISMA-P 2015 Checklist. A completed PRISMA-P checklist for the scoping review.

**Additional file 2.** Draft Search Strategy for Medline. A draft search strategy to be used in the Medline database.

## Data Availability

Not applicable

## Abbreviations

CNS: Central nervous system
OSF: Open Science framework
MeSH: Specific medical subject headings
