# Supplementary material for: Access to and experience of education for children and adolescents with cancer: a scoping review protocol
Source: Syst Rev. 2021 Jun 7;10:167. doi: 10.1186/s13643-021-01723-4 (PMC8182947; doi:10.1186/s13643-021-01723-4)
Supplement: Supplementary file 2 — Additional file 2. Draft Search Strategy for Medline. A draft search strategy to be used in the Medline database. [file 13643_2021_1723_MOESM2_ESM.docx]

**Population**

MeSH terms-exp Child, Adolescent

Key words –child*, schoolchild*, preschool*, pre school*, adolescent*, teen*, youth*, young person, young people.

**Concept**

MeSH terms- Education, Schools, Teaching, Education Distance, Schools, Nursery

Key words –education*, teach*, school*, hospital school*, re-entry program*, home school*, college*, virtual school*, virtual education, nursery school*, preschool*, pre school*, kindergarten*, secondary school*, primary school*, middle school*, high school*.

**Context**

MeSH terms-exp Neoplasms

Key words –neoplasm*, cancer*, malignancy, leuk?emia, brain tumo?r, central nervous system disease, CNS disease, germ cell tumo?r, embryonal tumo?r, AML, neuroblastoma, retinoblastoma, osteosarcoma, ewing* sarcoma, rhabdomyosarcoma, wilms tumo?r, Hodgkins lymphoma, non Hodgkins lymphoma, neuro-oncology, neurooncology, astrocytoma, low grade glioma, high grade astrocytoma, ependymoma, medulloblastoma, diffuse intrinsic pontine glioma, DIPG, Acute Lymphoblastic Leuk?emia, Acute Myeloid Leuk?emia.

Additional File 2: Draft Search Strategy for Medline
